# The silent healer: miR-205-5p up-regulation inhibits epithelial to mesenchymal transition in colon cancer cells by indirectly up-regulating E-cadherin expression

**DOI:** 10.1038/s41419-017-0102-8

**Published:** 2018-01-19

**Authors:** Diana Gulei, Lorand Magdo, Ancuta Jurj, Lajos Raduly, Roxana Cojocneanu-Petric, Alin Moldovan, Cristian Moldovan, Adrian Florea, Sergiu Pasca, Laura-Ancuta Pop, Vlad Moisoiu, Liviuta Budisan, Cecilia Pop-Bica, Cristina Ciocan, Rares Buiga, Mihai-Stefan Muresan, Rares Stiufiuc, Calin Ionescu, Ioana Berindan-Neagoe

**Affiliations:** 1 MEDFUTURE - Research Center for Advanced Medicine, “Iuliu-Hatieganu” University of Medicine and Pharmacy, Marinescu 23 Street / Louis Pasteur 4-6 Street, Cluj-Napoca, Romania; 2 Research Center for Functional Genomics, Biomedicine and Translational Medicine, ”Iuliu Hatieganu” University of Medicine and Pharmacy, 23 Marinescu Street, 400337 Cluj-Napoca, Romania; 3 Department of Pathophysiology, University of Agricultural Sciences and Veterinary Medicine, Calea Mănăștur 3-5 Street, 400372 Cluj-Napoca, Romania; 4 Department of Cell and Molecular Biology, Faculty of Medicine, “Iuliu Haţieganu” University of Medicine and Pharmacy, 6 Louis Pasteur St., 400349 Cluj-Napoca, Romania; 5 Faculty of Medicine, “Iuliu Hatieganu” University of Medicine and Pharmacy, 8 Victor Babes Street, Cluj-Napoca, Romania; 6 Department of Pathology, “Prof. Dr. Ion Chiricuta” Oncology Institute, Cluj-Napoca, Romania; 7 5th Surgical Department, Municipal Hospital, Cluj-Napoca, Romania; 8 Surgical and Gynecological Oncology Department, “Prof. Dr. Ion Chiricuta” Oncology Institute, Republicii 34 Street, 400015 Cluj-Napoca, Romania; 9 “Iuliu Hatieganu” University of Medicine and Pharmacy, Cluj-Napoca, Romania; 10 Department of Pharmaceutical Physics-Biophysics, Faculty of Pharmacy, “Iuliu Hatieganu” University of Medicine and Pharmacy, Pasteur 6 Street, 400349 Cluj-Napoca, Romania; 11 Department of Functional Genomics and Experimental Pathology, “Prof. Dr. Ion Chiricuta” Oncology Institute, Republicii 34 Street, Cluj-Napoca, 400015 Romania

## Abstract

EMT represents the dominant program within advanced stages of colon cancer, where cells acquire migratory characteristics in order to invade secondary tissues and form metastasis. Where the majority of the therapeutic strategies are concentrated on the reduction of the tumor mass through different apoptotic mechanisms, the present study advocates an important role for miR-205-5p in impairment of colon cancer cells migration and restoration of the epithelial phenotype. Upon identification of a homogenous downregulated profile for miR-205-5p in colon adenocarcinoma patients, functional studies demonstrated that experimental upregulation of this sequence is able to significantly raise the levels of E-cadherin through direct inhibition of ZEB1. Moreover, the elevation in CDH1 expression was translated into functional parameters where cells lost their invasion and migratory characteristics and formed homogenous clusters through adhesion interactions. Survival analysis of colon adenocarcinoma patients revealed that low levels of miR-205-5p are associated with an unfavorable prognostic compared to those with increased expression, demonstrating the possible clinical utility of miR-205-5p replacement. Exogenous administration of miRNA mimics was not associated with significant changes in cell viability or inflammatory pathways. Therefore, the proposed strategy is aiming towards inhibition of metastasis and limitation of the tumor borders in advanced stages patients in order to prolong the survival time and to increase the efficiency of the current therapeutic strategies.

## Introduction

Colon cancer ranks among the most common types of malignancies, occupying the third place in both men and women worldwide regarding new estimated cases and deaths^1^. Like in the case of most cancers, aggressive forms developed in late stages are frequently associated with low survival rates and an increased percent of mortality due to lack of effective treatment stratagems. Despite notable progresses in treatment of metastatic forms of colon cancer through incorporation of targeted biological agents (VEGF, VEGFR/multikinase, and EGFR inhibitors), a significant part of the late stages patients are characterized by an unresponsive phenotype^2–4^.

Epithelial to mesenchymal transition (EMT) is one of the central mechanism that stands at the base of metastasis, promoting the migratory phenotype of cancer cells through inhibition of adhesion molecules and stimulation of mesenchymal markers^5^. This transdifferentiation of epithelial cells towards mesenchymal ones allows the separation of transformed cells from the primary tumor and the migration towards secondary sites, contributing to the installation of metastasis^6,7^. The process is regulated by a number of key genes, which include the tumor suppressor CDH1 responsible for E-cadherin protein expression, Zinc Finger E-Box Binding Homeobox 1 (ZEB1) and SNAI1 (Zinc Finger Protein SNAI1), the two main suppressors of CDH1 and finally Vimentin (VIM), the principal biomarker of mesenchymal cells^8^. The reminded genes majorly manage the classical dynamics of EMT, but are in their turn regulated by microRNAs (miRNAs). These sequences inhibit the expression of target genes and also indirectly transpose their effects on the second line of transcripts.

miRNAs are short, non-coding sequences able to regulate gene expression through direct targeting of coding transcripts upon complementary hybridization^9,10^. The ability of these short sequences to inhibit the translation of tumor promoting or tumor suppressor genes is currently intensively explored in the oncology niche for tumor-targeted strategies^11–13^.

In the context of EMT modulation, both miR-200 family and miR-205 are markedly downregulated in cancer^14^, yet miR-200 group has captured most of the attention where all of the five members are proposed for targeted therapeutics^15,16^. Limited data is available for other miRNA sequences in respect to EMT impairment, particularly in colon cancer.

In the present study, we focused on miR-205-5p, sequence associated with tumor suppression features, but encountered as downregulated in colon cancer. Recent literature data associated this miRNA with ZEB1, a direct target, gene that in its turn inhibits the levels of E-cadherin in cancer cells, promoting the mesenchymal phenotype^14^. Although, this miRNA has been previously studied in several investigations, the potential of miR-205-5p to act as a targeted biological agent towards EMT inhibition in colon cancer is still not completely clarified. Moreover, the functional meaning of miR-205-5p was linked to the clinical scenario in order to gain knowledge about the possible role of the sequence as therapeutic tool in advanced forms of colon cancer.

## Results

### MiR-205 is frequently downregulated in colon cancer and associated with reduced survival among patients

**Fig. 1:**
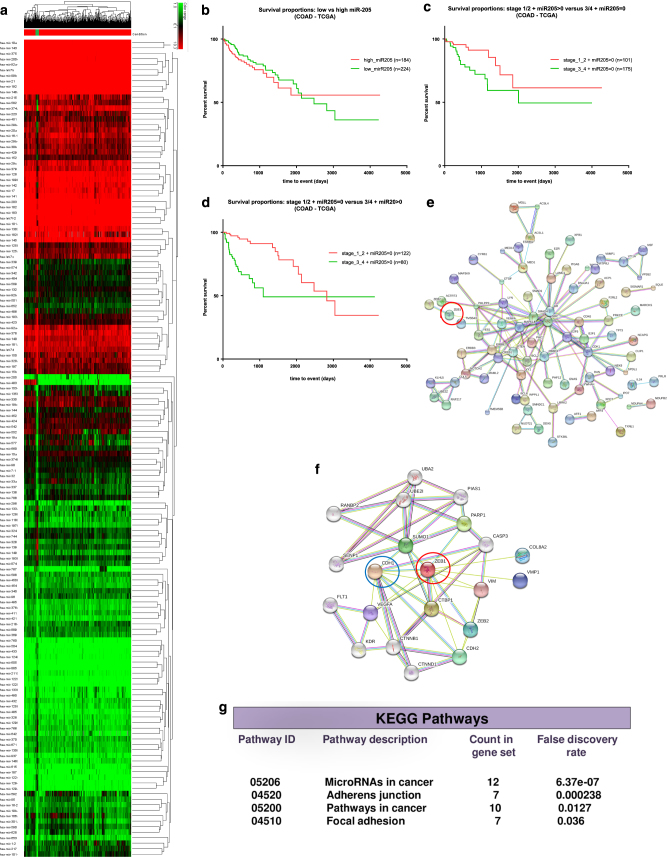
Expression of miR-205-5p in colon cancer patients and its prognostic value **a** Heatmaps of differential expressed miRNAs in colon cancer patients (*n* = 433) from TCGA reveals that miR-205 is constantly downregulated in the target pathology. **b** Kaplan–Meier analysis of survival of colon cancer patients with high miR-205 expression (*n* = 184) vs. low miR-205 expression (*n* = 224) extracted from TCGA indicates the prognostic value of miR-205. Survival time is given in days; *P* = 0.3637; *Χ*^2^ = 3.381. **c** Comparison between colon cancer patients with stage 1/2 and high miR-205 expression (*n* = 101) and colon cancer patients with stage 3/4 and low mir-205 expression (*n* = 175) in terms of survival percent in days indicate the significance of the miRNA sequence as a prognostic biomarker in terms of overall survival. Survival times is given in days; *P = 0.0158; *Χ*^2^ = 5.819. **d** Kaplan–Meier analysis of survival in colon cancer patients with stage 1/2 and low miR-205-5p (*n* = 122) expression and colon cancer patients with stage 3/4 with high miR-205 (*n* = 80) expression suggest a strong correlation of miR-205 with prolonged survival even in advanced stages of colon cancer. Survival times is given in days; ****P < 0.0001; *Χ*^2^ = 16.86. For all survival curves, data were extracted from TCGA data files and the separation of samples into low-expression and high-expression groups was conducted by using the median as cut-off. **e** Functional interactional network (generated by STRING analysis) of miR-205 targets and **f** ZEB1 targets indicate that miR-205 indirectly elevates the level of CDH1 (E-cadherin) through ZEB1 downregulation (miR-205 direct target). **g** KEGG pathways associated miR-205 predominantly with molecular adhesion mechanism in cancer based on the panel of the targeted genes

**Fig. 2:**
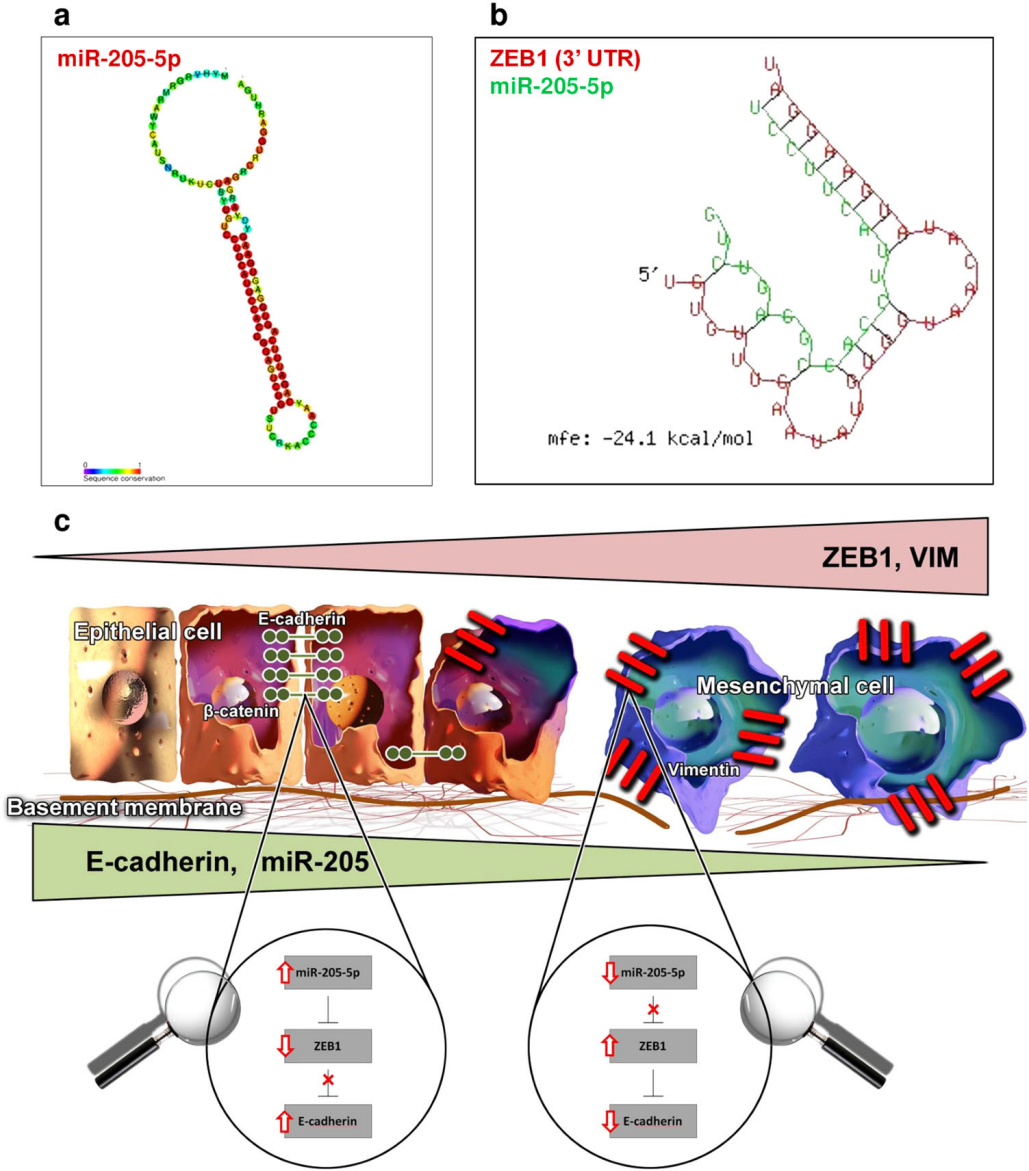
miR-205-5p in the context of cancer EMT **a** miR-205-5p **b** inhibits the transcriptional expression of ZEB1 through direct targeting of the 3′UTR region (mfe: −24.1 kcal/mol—generated with BibiServ—RNAhybrid^29^). In its turn, ZEB1 is one of the main repressor of CDH1 (E-cadherin). **c** Therefore, high levels of miR-205 are associated with an epithelial phenotype (increased E-cadherin), where low levels of the same sequence determine the transition towards a mesenchymal one (increased VIM)

Based on expression profiles from 433 colon adenocarcinoma (COAD) patients from TCGA database, miR-205 was found as frequently downregulated compared to normal colon tissue, presenting a homogenous pathological profile (Fig. 1a). Following this pattern, we next determined the association of miR-205 with overall survival of colon cancer patients (Fig. 1b). According to the expression levels of the miRNA sequence it was found a negative correlation between miR-205 downregulation and time of survival within colon cancer patients (*P* = 0.3637; *Χ*^2^ = 0.8250). Cases with high miR-205 levels presented a better prognostic value. The same trend was constant in terms of overall survival between colon cancer patients with stage 1/2 and high miR-205 expression and patients with stage 3/4 and low miR-205 expression (*P = 0.0158; *Χ*^2^ = 5.819) (Fig. 1c). More significantly, Kaplan–Meier analysis showed a marked increased survival among patients with stage 3/4 and high levels of the target miRNA against patients with incipient stages, but low expression of miR-205 (****P < 0.0001; *Χ*^2^ = 16.86) (Fig. 1d). Analysis of human miR-205-5p validated targets (Supplementary Table 1) using miRWalk database and generation of gene network interactions using STRING analysis (Fig. 1e, f) showed ZEB1 as one of the main targets of miR-205-5p. ZEB1 directly downregulates CDH1, a key gene for cell adhesion mechanisms. KEGG pathway confirmed this proposed correlation, where miR-205 is mainly involved, among other functional pathways, in adherens junction and focal adhesion mechanisms (Fig. 1g). Based on these data, we hypothesized that miR-205-5p overexpression was requested in order to restore the epithelial phenotype of colon cancer cells and implicit impair the migratory characteristics associated with EMT, by slowing down its evolution (Fig. 2).

### MiR-205-5p is downregulated in colon cancer patients, as well as in HCT-116 and RKO cell lines, where exogenous administration of miRNA mimic significantly raises the level of the sequence in both cell lines

**Fig. 3:**
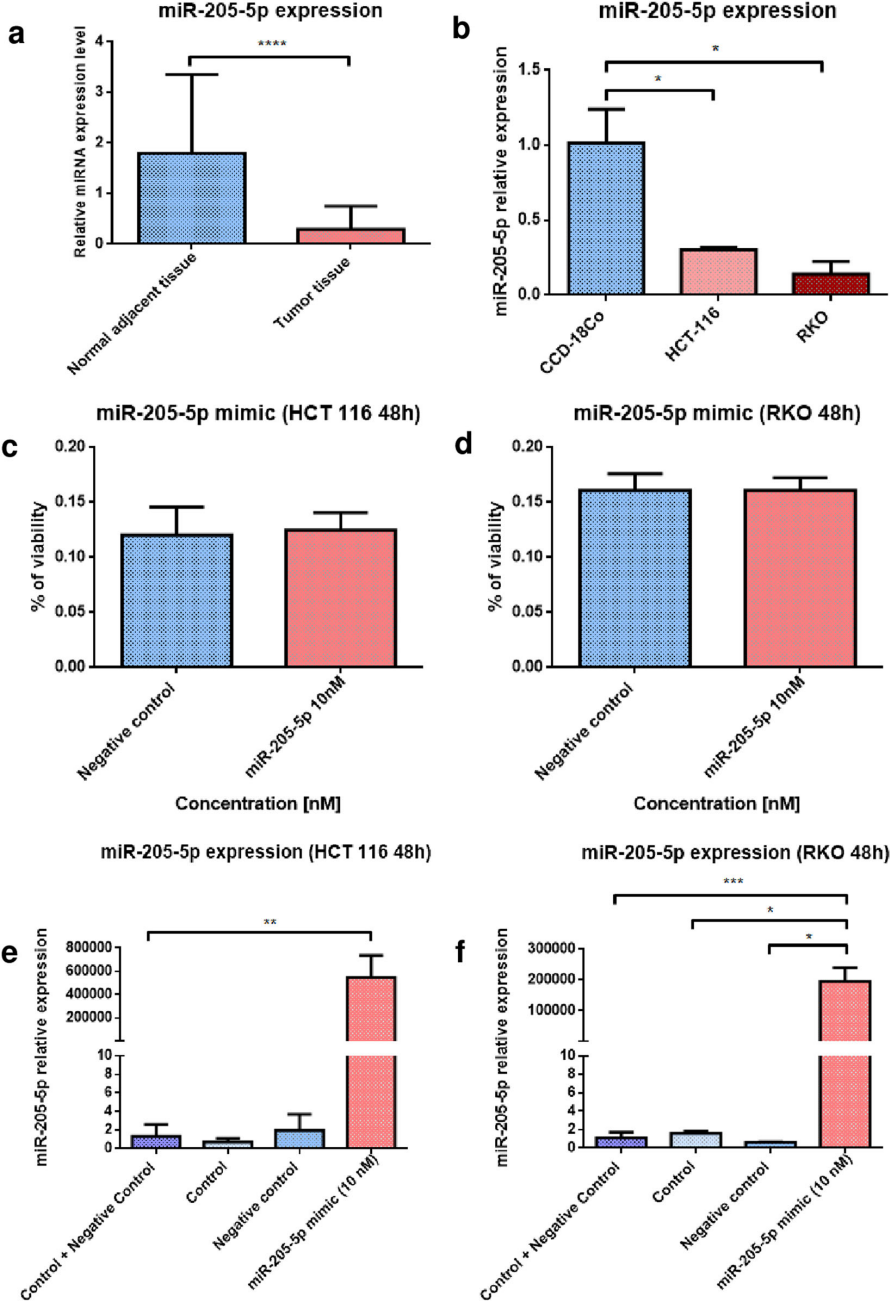
miR-205-5p expression and modulation in HCT-116 and RKO cell lines **a** RT-qPCR analysis of colon cancer tissue samples (*n* = 23) compared with adjacent normal samples (*n* = 15) showed that miR-205 is significantly downregulated in patients diagnosed with colon cancer (data presented as mean ± S.D.; ****P < 0.0001, independent *t*-test compared to control). Experiments were performed in duplicate for each sample and miR-205-5p relative expression was expressed by fold-change and normalized to U6 snRNA. **b** RT-qPCR analysis of HCT-116 and RKO colon cancer cell lines revealed that miR-205-5p is significantly downregulated compared to the normal colon cell line (CCD-18Co) (data presented as mean ± S.D.; *P = 0.0477 for HCT-116, independent *t*-test compared to CCD-18Co, and *P = 0365 for RKO independent *t*-test compared to CCD-18Co). Experiments were performed two times in duplicates for each cell line and miR-205-5p relative expression was expressed by fold-change and normalized to U6 snRNA. Cell viability assay (MTT) revealed no significant toxicity of exogenous miR-205-5p (10 nM) expression in **c** HCT-116 and **d** RKO cells (48 h post transfection). Cells were transfected with either human miR-205-5p mimic or negative control using Lipofectamine 2000. Experiments were performed three times. Real-time PCR analysis of transfected **e**, HCT-116 cells (data presented as mean ± S.D.; **P = 0.0027, independent *t*-test compared to control and negative control) and **f** RKO cells (data presented as mean ± S.D.; ***P = 0.0005, independent *t*-test compared to control and negative control; *P = 0.0245, independent *t*-test compared to control and *P = 0.0245, independent *t*-test compared to negative control) with miR-205-5p mimic for 48 h indicate that the level of the miRNA sequence was significantly upregulated compared with control samples. Modifications in miR-205-5p expression are represented as grouped data graph were on the *y*-axis was plotted as the differential expression in fold-change of miRNA sequence compared to control. Experiments were performed two times in duplicate for each sample

Data from TCGA regarding miR-205 expression was confirmed through RT-qPCR examination on a cohort of 30 COAD patients with stage 3/4 prior to any chemotherapy and not associated with high-risk hereditary mutations (Table 1) from The “Ion Chiricuta” Oncology Institute and County Hospital, Cluj-Napoca. Therefore, miR-205-5p was found significantly downregulated in tumor tissue samples compared to normal adjacent ones with a statistical difference of ****P < 0.0001 (Fig. 3a). In order to evaluate if HCT-116 and RKO colon cancer cell lines follow closely the miR-205-5p downregulated pattern found in colon cancer patients, qRT-PCR analysis was performed for the two cell lines compared to a normal colon cell line (CCD-18Co) (Fig. 3b). Both lines revealed a significantly downregulated profile for miR-205-5p, with a slight increase in HCT-116 cells compared to RKO. Exogenous expression of miR-205-5p was obtained upon transfection with a minimum dose of 10 nM of miR-205-5p mimic (mirVana) using Lipofectamine 2000. The dose was chosen to closely mimic the homeostatic situation within normal cells and not cause any significant undesired adverse effects. MTT assays at 48 h post transfection showed no significant differences in cell survival (Fig. 3c,d), demonstrating that the dose of miR-205-5p sequence does not stress any apoptotic pathways or induce toxic effects following miRNA transfection. RT-qPCR analysis of the two cell lines confirmed the elevated levels of miR-205-5p in both treated cells compared to controls, the increase being more prominent in HCT-116 (**P = 0.0027) (Fig. 3e) besides RKO cell line (***P = 0.0005 control and negative control; *P = 0.0245 control and *P = 0.0245 negative control) (Fig. 3f) consequent to transfection with identical miR-205-5p doses (10 nM).

**Table 1:** Clinical characteristics of human subjects

Characteristic	*N* = 30
Age	
50–60	12
60–70	8
70–80	8
>80	2
Gender	
Male	15
Female	15
TNM	
T3	25
T4	5
N0	15
N1	6
N2	9
M0	25
M+	5
Neoadjuvant therapy	
Yes	0
No	30
Hereditary mutations	
Yes	0
No	30

### MiR-205-5p upregulation significantly elevates the levels of CDH1 in HCT-116 cell line through downregulation of ZEB1 expression

**Fig. 4:**
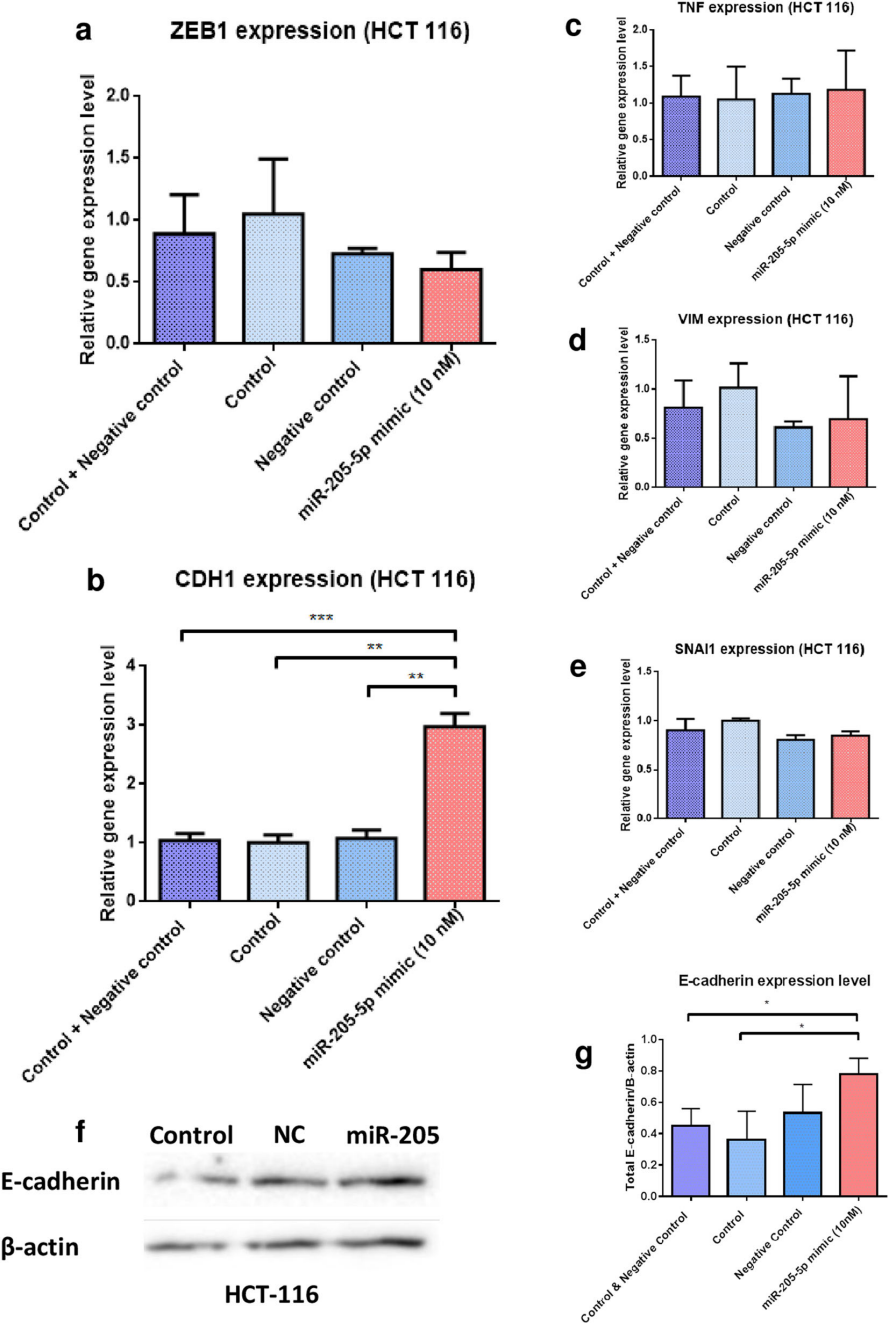
Exogenous expression of miR-205-5p in colon cancer cells regulates the expression of genes involved in EMT HCT-116 colon cancer cell line was transfected with either 10 nM human miR-205-5p mimic or negative control (mirVana) using Lipofectamine 2000 and expression values of target and additional genes were analyzed 48 h post-transfection by RT-qPCR. All values were normalized to HPRT-1 and RPLP0 housekeeping genes and represented in grouped data graph were on the *y*-axis, was plotted the differential expression in fold-change of the analyzed genes compared to control samples. **a** RT-qPCR analysis of ZEB1 mRNA expression in HCT-116 cell lines indicated that upregulation of miR-205-5p inhibits the levels of the target gene compared with control, negative control, and combination between the two sets of values (data presented as mean ± S.D). Experiments were performed two times in duplicate for each cell line. **b** RT-qPCR for CDH1 expression in HCT-116 cells transfected with miR-205-5p mimic showed a significantly elevated level of the CDH1 gene compared to controls (data presented as mean ± S.D.; ***P = 0.0001, independent *t*-test compared to control and negative control; **P = 0.0083, independent *t*-test compared to control and **P = 0.0092, independent *t*-test compared to negative control). Experiments were two times in duplicate for each cell line. RT-qPCR analysis of additional regulatory genes with impact on inflammation: **c** TNF and EMT: **d** VIM and **e** SNAI1 in HCT-116 cell line transfected with miR-205-5p indicated no significant changes in the expression profile compared to controls. **f** WB for E-cadherin (120 kDa) indicates that upregulation of CDH1 upon miR-205-5p transfection was preserved also at the translational level in HCT-116 cells with a significant elevation in E-cadherin protein expression. Experiments were performed in triplicates. **g** Quantification of WB for E-cadherin protein expression shows that miR-205-5p transfection significantly elevated the protein levels compared to controls (data presented as mean ± S.D.; *P = 0.0201, independent *t*-test compared to control and negative control; **P = 0.262, independent *t*-test compared to control)

Next, it was assessed the functional meaning of miR-205-5p upregulation in the two cell lines by analyzing the expression values of ZEB1, the direct target gene of the miRNA sequence, and also other additional regulatory elements. Based on the previous direct association of miR-205-5p and ZEB1, RT-qPCR analysis on HCT-116 colon cancer cells exhibit a decrease in mRNA levels compared with the control counterparts 48 h after miR-205-5p (10 nM) exogenous upregulation (Fig. 4a). The effect was propagated on the status of CDH1, with a significant increase in expression by approximately a fold-change of 3 (***P = 0.0001 control and negative control; **P = 0.0083, control and **P = 0.0092 negative control) (Fig. 4b). These data reveal that by targeting ZEB1, a promoter of the EMT, enforced levels of miR-205-5p are indirectly acting on the expression of CDH1, a central gene in the preservation of the normal epithelial phenotype and impairment of cancer cells invasiveness. Additionally, there was no significant difference in the levels of TNF mRNA, suggesting that the selected dose of miRNA is not altering TNF levels of expression, this gene being well known as a central regulator of inflammation (Fig. 4c). Vimentin, a marker of the mesenchymal phenotype was also unassociated with difference in transcriptional expression values (Fig. 4d), the same as in the case of SNAI1, another promoter of the EMT through CDH1 suppression (Fig. 4e). All together, these last two sets of results dissociates miR-205-5p from the regulatory pathways related to EMT inhibition obtained through suppression of Vimentin or SNAI1 and confirms the regulatory pathway through ZEB1 downregulation that indirectly generates elevated levels of CDH1. To confirm the functional expression of CDH1 within transfected cells (HCT-116), WB analysis was performed in triplicate using monoclonal antibodies for E-cadherin and, as expected, there was a marked difference between protein levels found in control, negative control and miR-205 transfected cells (Fig. 4f,g, Supplementary Fig. 1). QRT-PCR and WB analyses in RKO cell line were shown a different pattern (data presented in Supplementary Fig. 2), underlying the tumor heterogeneity even in the same adenocarcinoma histological type.

### MiR-205-5p by itself affects colon cancer cell phenotype impairing the migratory characteristics and promoting the restoration of adhesion between treated cells

**Fig. 5:**
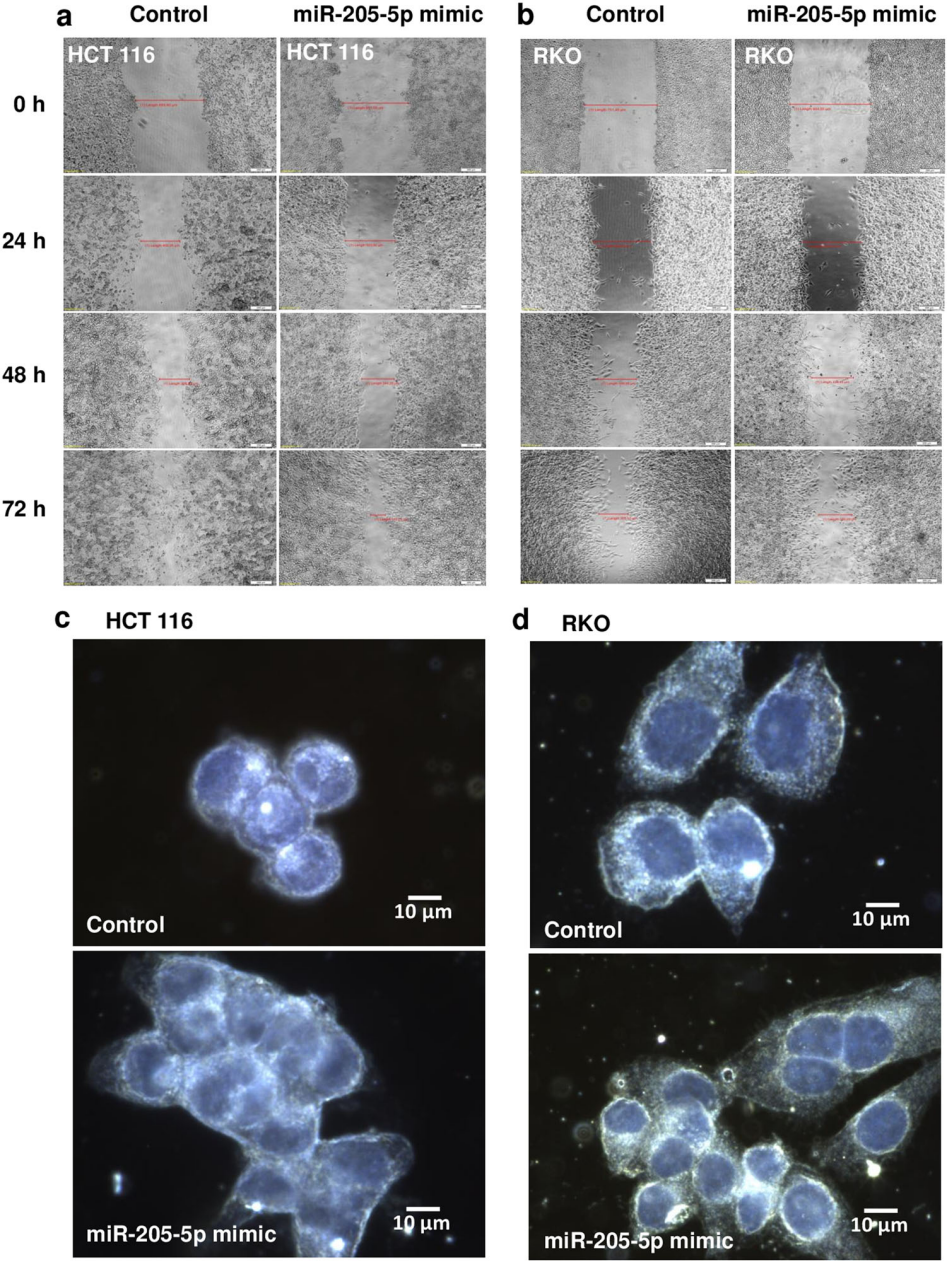
miR-205-5p experimental overexpression impairs the migratory phenotype of colon cancer cells and enhances the adhesion between cells **a** Wound healing assay for cell motility was performed on HCT-115 and RKO colon cancer cells for both transfected (10 nM miR-205-5p mimic, 48 h) and control samples in different time points. For HCT-115 overexpressing miR-205-5p difference were marked beginning with 24 h and ending with 72 h where the gap was closed in control samples and incompletely seal (157.25 µm) in the experimental group. RKO cell line was not associated with significant difference between control and miR-205-5p overexpression cells in terms of migration capacity. Experiments were performed two times. **b** Differences in cell adhesion were analyzed by ultraspectral imaging by 60× or 100× resolution using immersion fluorite objectives and a triple-pass filter configuration. Darkfield images indicate a predisposition of the treated cells to adhere to each other and form clusters compared to control samples where cells were observed as more individually distributed. Constant with healing assay, HCT-116 cells transfected with miR-505-5p mimic revealed a more potent modification in terms of focal adhesions

In order to assess the functional modifications related to miR-205-5p overexpression, wound healing assay was performed at different time points for both HCT-116 (Fig. 5a) and RKO (Fig. 5b) colon cancer cells lines 48 h after transfection with 10 nM of miR-205-5p mimic. In the case of the first cell line, the decisive time point of 72 h marked the complete closure of control cells, while in the treated ones a gap of 157.25 µm was still shown. These data suggest that the elevated levels of E-cadherin at transcriptional and translational levels are associated with a functional effect, where treated cells are less prone to invasion and implicit metastasis. Dark field images of HCT-116 line obtained through ultraspectral analysis confirmed the phenotype of experimental cells, where in the case of the miR-205-5p treated group it was observed as clusters of associated cells that were interconnected through adhesion points (Fig. 5c). The control counterparts were more dissociated and also characterized by a more individualized pattern. In the case of RKO colon cancer line, the difference was not as significant, although modest modifications in cell distribution could be seen (Fig. 5d) with a predisposition of clustering into groups of cells.

### Inverse correlation of E-cadherin and Vimentin markers upon miR-205-5p transfection coupled with adherens junctions between cells

**Fig. 6:**
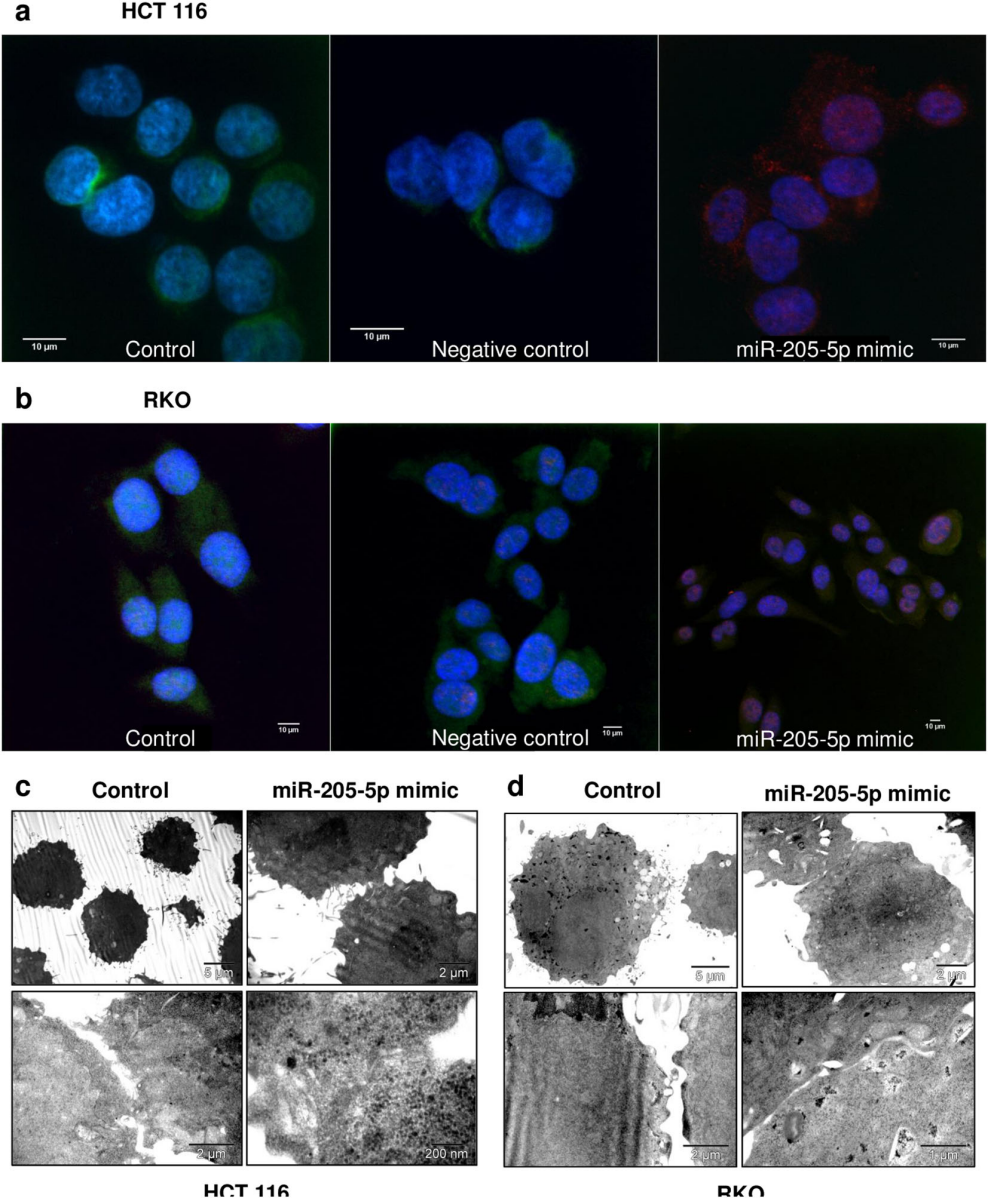
miR-205-5p enforced expression promotes the epithelial phenotype and adherens junctions between colon cancer cells Immunostaining analysis of **a** HCT-116 and **b** RKO colon cancer cells marked with primary antibody for Human E-cadherin (secondary antibody conjugated with DyeLight549) and Vimentin (secondary antibody conjugated with FITC) was used for detection of the target proteins in controls and treated cells (10 nM miR-205-5p mimic, 48 h) and DAPI for nucleus staining. Each experiment was performed independently for two times. Ultrathin sections of **c** HCT-116 and **d** RKO control and transfected cells (10 nM miR-205-5p mimic, 48 h) were analyzed by TEM for ultrastructural modifications upon miR-205-5p treatment. In both IF and TEM experiments, HCT-116 cells revealed significantly increased expression of E-cadherin and low expression of Vimentin compared with the control counterparts, while RKO cell line was associated with a more moderate response

Epithelial cells are usually associated with increased expression of E-cadherin and low expression of Vimentin, while transformed mesenchymal cells are correlated with the inverse proportions of the two markers. After miR-205-5p transfection (10 nM) experimental cells and associated controls were stained with antibodies directed towards E-cadherin and Vimentin in order to assess the relative expression, disposition, and preponderance of the two molecules within the colon cancer cells. HCT-116 control samples were characterized by increased levels of Vimentin and implicit a mesenchymal/mesenchymal-like phenotype, while miRNA treated group was associated with potentiated E-cadherin on their surface (Fig. 6a). Therefore, miR-205-5p alone in a limited dose of 10 nM was able to significantly modify the phenotype of cancer cells and impair the EMT hallmarks. Modifications in RKO cell lines: control vs. miRNA-205-5p mimic revealed a restricted increase in E-cadherin and decrease in Vimentin, suggesting a possible miRNA dose-related mechanisms or interference with other regulatory pathways (Fig. 6b). Also, the proportions of Vimentin in RKO and HCT-116 control cells was observed as faintly different with a more potentiated expression in RKO, suggesting a more invasive and aggressive behavior of these cells. Ultrastructural modifications were followed performing ultrathin sections of the treated cells (miR-205-5p mimic 10 nM, 48 h) previously embedded in resin and analyzed through TEM. For HCT-116 line, control cells were observed to follow a dispersed pattern, while the treated ones came in close contact (Fig. 6c). Detailed analysis of the adjacent cell membranes marked the existence of adhesion junctions, characteristics of epithelial cells, while in the case of the control ones, the cells and implicit the membranes were distanced from each other with no functional junctions. No additional modified ultrastructural features were observed and also the percent of viability was of 100%. In the case of the RKO cell line, a similar structural pattern was shown, but not as pronounced as in HCT-116 cells (Fig. 6d). Although the modifications were not as conclusive, there was a differences between miR-205-5p group and control samples where cells had a slight tendency to come in contact, but without firm adhesions.

### Colon cancer patients follow closely the in vitro trend in terms of CDH1 and ZEB1 expression

**Fig. 7:**
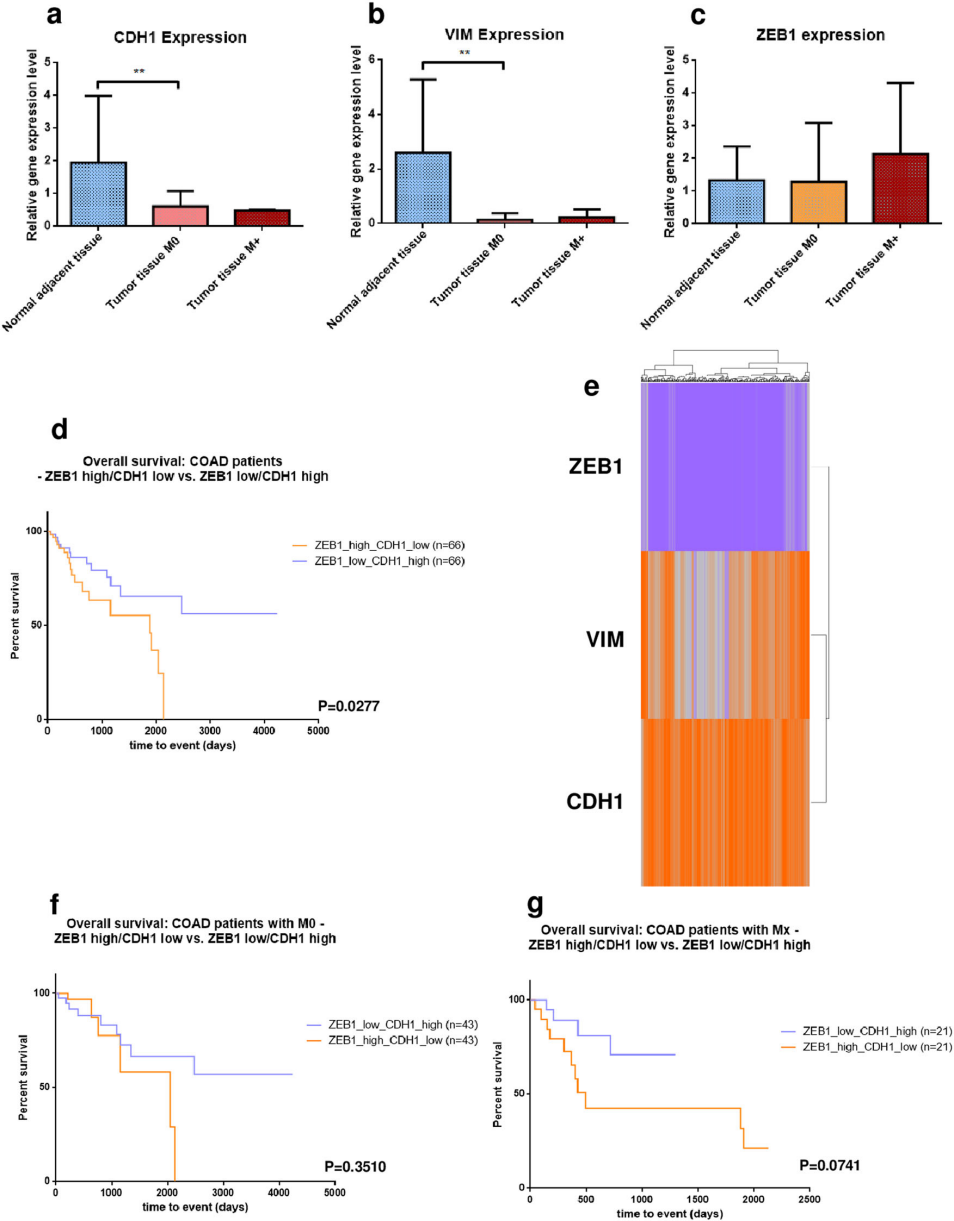
CDH1 combined with ZEB1 expression is a valuable prognostic biomarker in colon cancer patients RT-qPCR analysis of colon cancer tissue samples compared with adjacent normal samples demonstrates a role of CDH1 and ZEB1 expression as prognostic biomarkers. All values were normalized to HPRT-1 housekeeping gene and represented in grouped data graph were on the *y*-axis was plotted the differential expression in fold-change of the analyzed genes compared to control samples. Tumor samples were obtained from The “Ion Chiricuta” Oncology Institute and County Hospital, Cluj-Napoca and divided according to the presence/absence of metastasis. **a** CDH1 expression was verified by RT-qPCR in tumor tissue from patients with M0 (*n* = 16) and M+ (*n* = 2) against normal adjacent tissue (*n* = 11). CDH1 expression was found significantly lower in malignant samples M0 (data presented as mean ± S.D.; **P = 0.0094, independent *t*-test compared to control) and even more deregulated in patients with metastasis. All experiments were performed in duplicate. **b** RT-qPCR demonstrated decreased expression of Vimentin in colon cancer samples (data presented as mean ± S.D.; **P = 0.0094, independent *t*-test compared to control) with a slight upregulation in M+ (*n* = 2) vs. M0 (*n* = 12) to normal adjacent tissue (*n* = 6) (data presented as mean ± S.D.). All experiments were performed in duplicate. **c** ZEB1 expression was upregulated in malignant samples with a marked increased in M+ (*n* = 2) as against both M0 (*n* = 16) and normal tissue (*n* = 7). All experiments were performed in duplicate. **d** Kaplan–Meier analysis of survival between colon cancer patients from TCGA with high ZEB1 and low CDH1 expression (*n* = 66) and low ZEB1 and high CDH1 expression (*n* = 66) indicate a possible prognostic role for the combination between the inversely proportional expressed biomarkers. Survival times are given in days; *P = 0.0277; *Χ*^2^ = 4.847. **e** Heatmap of differentially expressed genes in colon cancer cases from TCGA demonstrates the inverse correlation between ZEB1 and CDH1 expression. Kaplan–Meier analysis for survival for **f** colon cancer patients with M0 (n-high ZEB/low CDH1 = 43, n-low ZEB/high CDH1 = 43; *P* = 0.3510; *Χ*^2^ = 0.8699) and **g** colon cancer patients with M+ (n-high ZEB/low CDH1 = 21, n-low ZEB/high CDH1 = 21; *P* = 0.0741; *Χ*^2^ = 3.190) shows that combination of high ZEB1 and low CDH1 expression is associated with a more lethal phenotype regardless of the non-metastatic/metastatic profile. Survival times is given in days and the separation of samples into low-expression and high-expression groups was conducted by using the median as cut-off

EMT is considered a crucial step in metastasis and is predominantly encountered in patients with advanced stages, where malignant cells acquire migratory characteristics and invade secondary sites. In order to validate the inhibition of ZEB1 on CDH1 upon EMT, we examined by RT-qPCR a cohort of 30 colon adenocarcinoma patients with stage 3/4 prior to any chemotherapy and not associated with high-risk hereditary mutations (Table 1). Stages 3 and 4 were classified according to their metastatic status in M0 (no metastasis) and M+ (with metastasis). Expression values of CDH1 in tumor samples (Fig. 7a) were significantly decreased compared to the adjacent normal tissue (**P = 0.0094 normal adjacent tissue vs. tumor tissue M0) suggesting that tumor cells within the samples are losing their adherence capacity and acquire invasive potential. Moreover, a marked difference was also observed when comparing the levels of CDH1 expression between M0 and M+ patients. VIM expression followed a similar trend (Fig. 7b) in respect to the transcriptional values (**P = 0.0094 normal adjacent tissue vs. tumor tissue M0). As expected, the levels of ZEB1, a potent inhibitor of CDH1 expression, were inversely correlated with the expression of the target gene, where M0/M+ patients were characterized by high amounts of ZEB1 mRNA (Fig. 7c). Kaplan–Meier analysis of survival between colon cancer patients with increased ZEB1 and decreased CDH1 expression vs. patients with low ZEB1 and high CDH1 values promotes the possible prognosis value for the combination between the two genes (Fig. 7d). Therefore, the first group of patients was associated with an unfavorable pattern of survival (*P = 0.0277; *Χ*^2^ = 4.847), while in the second group the time of survival was significantly prolonged. Heat maps of 288 COAD samples generated based on the expression of ZEB1, VIM, and CDH1 accentuated the inverse correlation between ZEB1 and CDH1, while VIM had a larger segment of expression variation (Fig. 7e). Collectively, these results indicate that the combination between the two antagonist genes (CDH1 and VIM) could represent a valuable prognostic marker in colon cancer. Moreover, differential analysis in survival of M0 (*P* = 0.3510; *Χ*^2^ = 0.8699) (Fig. 7f) and M+ (*P* = 0.0741; *Χ*^2^ = 3.190) (Fig. 7g) patients showed that the inversely correlated expression pattern of the two target genes (high ZEB1 and low CDH1) was associated with a disadvantageous prognostic regardless of the metastatic profile. In these terms, inhibition of ZEB1 and subsequent upregulation of CDH1 becomes an advantageous strategy especially in the case of patients with advanced stages of colon cancer. We also performed some additional survival combinations (Supplementary Fig. 3) that involves the association between miR-205 and CDH1 or VIM expression levels. The results show that combination between high levels of miR-205 and increased expression of CDH1 is the best scenario in terms of survival rates for colon cancer patients representing the intention of the present therapeutic strategy.

## Discussions

Dysregulation of miR-205 in colon cancer has been studied in different histopathological entities, being predominantly associated with tumor suppressor functions through targeting of oncogenes involved in the main cancer hallmarks^17–21^. In terms of colon cancer the expression of miR-205 is dependent on the specific subtype^18^. Despite colon cancer heterogeneity, it seems that one of the dominant program within the targeted pathology is EMT, with a pattern of dysregulated genes that are constantly associated with implications in one of the two phenotypes: epithelial or mesenchymal^22^. While miR-200 family has been extensively studied as potent inhibitor of the phenotypically switch, a far less number of studies investigated the possible role of miR-205 in reversing the invasive potential of transformed cancer cells^7^.

Analysis of COAD patients from TCGA database revealed that miR-205 is downregulated in this pathology, where the survival percent associated with low expression of the miRNA sequence are decreased compared with the high expression group. Moreover, KEGG analysis identified two separate pathways associated with cell adhesion based on miR-205 target genes, fact that outlined the hypothesis of the present study, where effects of experimental upregulation of miRNA were tested in the context of EMT inhibition.

Transfection of miR-205-5p in colon cancer cells induced low levels of ZEB1, a direct target of the non-coding sequence, results that overlap with data from previous studies^14,23^. ZEB1 is recognized as a repressor of CDH1 levels with implicit negative effects on the preservation of the epithelial phenotype^24^. In order to verify the ability of miR-205 to indirectly increase the expression of E-cadherin gene we performed RT-qPCR analysis and found significantly greater levels of the tumor suppressor gene, reveling that miR-205 alone is able to restore the epithelial phenotype of cancer cells. No significant changes in SNAI1 were observed, gene that has a strong suppression action upon E-cadherin levels, demonstrating the potentiality of miR-205 to abrogate the inhibitory pattern. Similar results were obtained for VIM transcriptional levels, a mesenchymal marker, where no important differences were found.

Functional wound assay on treated cells further confirmed that E-cadherin reinforcement is altering the migratory character of target cells. Immunostaining and ultrastructural analysis strengthen the proposed hypothesis, where cells expressed functional adhesion proteins and were grouped in clusters based on the interactions between cell membranes. As important, consistent with the initial results from the MTT assay, miR-205 did not affect the viability of the transfected cells and no apoptotic features were observed on TEM visualization. Collectively, these results show that miR-205 replacement alone is able to impair the mesenchymal phenotype of colon cancer cells, slowing the migratory characteristics.

In order to verify that the present therapeutic strategy has a notable value in colon cancer patients, a local cohort of tumor samples from individuals diagnosed with colon cancer, stages 3/4, was analyzed for CDH1, VIM, and ZEB1 expression, these three genes representing the most important markers of EMT^25^. As expected, RT-qPCR individualized an invasive and metastatic profile in the analyzed samples, where low expression of CDH1 and high levels of ZEB1 were constantly shown. Moreover, the profile of these two key regulatory genes was differentially altered in patients with and without metastasis. Survival curves based on ZEB1/CDH1 expression sustained the important role of the two genes in colon cancer prognosis, where combination between increased ZEB1 expression and decreased CDH1 levels significantly reduced the time of survival.

In summary, the present study demonstrated the important role of miR-205 alone in suppression of EMT and restoration of the epithelial phenotype of in vitro colon cancer cells through ZEB1 targeting and CDH1 upregulation. The presented results sustain a possible value of miR-205 in cancer therapeutics, where impairment of cancer invasiveness will significantly prolong the time of survival within advanced cancer patient (where treatment options are limited) and also will keep in line the primary tumor for further therapeutic strategies.

## Materials and methods

### Clinical tissue samples and cell cultures

COAD and normal adjacent tissue samples were collected from patients from The “Ion Chiricuta” Oncology Institute and County Hospital, Cluj-Napoca, Romania. All patients signed an informed consent approved by the Ethical Committee. Samples were collected during surgery and then stored in liquid nitrogen upon further experimental analysis. All samples were accompanied by patients’ clinical data. Two colon carcinoma cell lines, RKO and HCT 116, were cultured in McCoy’s 5A medium with 10% fetal bovine serum (FBS) at 37 °C with 5% CO_2_. The normal colon cell line, CCD-18Co, was cultured in EMEM medium with 10% fetal bovine serum (FBS) at 37 °C with 5% CO_2_.

### Bioinformatics analysis

The TCGA matrices containing gene and microRNA expression data—normalized and in logarithmic format—for COAD samples were downloaded from the University of California Santa Cruz Cancer Browser^26^, together with the respective clinical data files, which, among other materials, contain clinical and survival information. Differential expression was performed with the help of the Gene Spring GX software (Agilent Technologies), using a *P*-value < 0.05, and a fold change threshold of 2.0 in the case of genes, and 1.5 for miRNA. The graphical representation via Heatmaps was obtained using the same software application. The data matrices were also used to extract expression data for miR-205-5p, ZEB1, CDH1 and VIM, together with the survival information from the clinical data files, in order to perform Kaplan–Meier curves using the Graph Pad Prism software (Graph Pad Software, Inc). The separation of samples into low-expression and high-expression groups was conducted by using the median as cut-off. Regarding the identification of target genes for the studied miRNAs, the used tool was the validated module from the miRWalk 2.0 platform^27^, the downloaded files being further processed using Excel (Microsoft Office)—sorting, elimination of duplicates, etc. The interactions between these genes were predicted with the help of the String DB online platform^28^, taking into consideration only the high confidence interactions (interaction score: 0.7), and displaying only the proteins which showed direct connections (by checking the “hide disconnected nodes in the network” option).

### Hsa-miR-205-5p and negative control transfection

RKO and HCT-116 transient transfections were performed using Lipofectamine 2000 (Invitrogen) according to the manufacturer’s instructions. Hsa-miR-205-5p mimic (Ambion) and negative control (Ambion) diluted in Opti-MEM Medium (Gibco) were mixed and incubated at room temperature for 5 min and added to the cells. After 6 h the medium was aspirated and replaced with McCoy’s 5A medium with 10% FBS. Forty-eight hours post-transfection the cells were ready for functional evaluation. As controls were considered the following situations: “control”, where cells did not receive any type of treatment; “negative control”, where cells were transfected with miRNA mimic negative control and finally, “control + negative control” that includes the combined values from both “control” and “negative control” groups.

### Cell viability assay

For cell viability assay, RKO and HCT-116 cells were seeded at a density of 8 × 10^3^ cells per well in 96-well plates. Cells were transfected with hsa-miR-205-5p and negative control at 0 h. At 48 h from the plating, the medium was aspirated and 150 µl thyazolyl blue tetrazolium bromide (1 mg/ml, Sigma, USA) was added for 1 h at 37 °C. After thyazolyl blue tetrazolium bromide removal, cells were dissolved in 100 µl DMSO. The absorbance was measured after 10 min using a plate reader.

### RNA isolation and RT-qPCR

Total RNA from cells and tissue samples was extracted using TriReagent (Ambion) based on the producer recommended protocol. RNA concentrations and quality were assessed using NanoDrop-1100 by measuring the absorbance of UV–Visible light. Sample purity was assessed based on the spectral data and purity ratios. Total RNA (50 ng for miRNA expression and 500 ng for gene expression/sample) was reversed transcribed into cDNA using TaqMan MicroRNA Reverse Transcription Kit (Applied Biosystems) for miRNA expression analysis and High Capacity cDNA Reverse Transcription Kit (Applied Biosystems) for gene expression evaluation. For miRNA amplification we used SsoAdvanced Universal Probes Supermix (Bio-Rad) and SYB Select Master Mix for gene expression. RT-qPCR was performed with ViiA™ 7 System in a 5 µl (miRNA amplification) and 10 µl (gene amplification) volume using a 384-well plate (cDNA dilution—1:5). The primer sequences were as follows:

SNAI1 left: 5′ GCT GCA GGA CTC TAA TCC AGA 3′

SNAI1 right: 5′ ATC TCC GGA GGT GGG ATG 3′

ZEB1 left: 5′ AAC TGC TGG GAG GAT GAC AC 3′

ZEB1 right: 5′ TCC TGC TTC ATC TGC CTG A 3′

VIM left: 5′ TGG TCT AAC GGT TTC CCC TA 3′

VIM right: 5′ GAC CTC GGA GCG AGA GTG 3′

CDH1 left: 5′ GGT CTG TCA TGG AAG GTG CT 3′

CDH1 right: 5′ GAT GGC GGC ATT GTA GGT 3′

TNF left: 5′ CAGCCTCTTCTCCTTCCTGAT 3′

TNF right: 5′ GGGGAACTCTTCCCTCTG 3′

### Wound healing assay

Wells covered in treated and control cells from a 24-well plate were scratched with a 20 µl pipette. Wound closure was observed and photographed at 0, 8, 24, 48, and 72 h.

### Western blot

Cell lysate in equal concentrations were resolved through electrophoresis in sodium dodecylsulfate polyacrylamide gel (100 V through stacking gel and 200 V for resolving gel) and then transferred onto a nitrocellulose membrane (Bio-Rad). After blocking, membrane was incubated o/n with E-cadherin primary antibody (Monoclonal Mouse IgG_2B_ #180215) diluted in non-fat milk, washed with TBST for three times and then incubated with the HRP-conjugated secondary antibody. After three washing steps, the membrane was incubated at RT for 5 min with chemiluminescent substrate (SuperSignal West Pico, Thermo Scientific). Protein expression was visualized using Azure C300 Instrument (Azure Biosystems) in the chemiluminescent mode and quantification was done with ImageJ Software.

### Immunocytochemistry and dark-field imaging

RKO and HCT 116 cells were cultured in eight-well chamber slides (75,000 cells per well). Cells were transfected with hsa-miR 205-5p and negative control at 0 h. At 48 h from plating, culture media was aspirated, then the cells were covered with paraformaldehyde (4%) for 15 min at room temperature, after which the paraformaldehyde was aspirated and cells were washed two times with 400 µl of wash buffer. Four hundred microliters of blocking buffer were added and the chamber slides were incubated at room temperature for 45 min, after which blocking buffer was removed. Monoclonal rat Vimentin primary antibody and monoclonal mouse E-Cadherin primary antibody were applied and incubated at room temperature for 1 h. Chamber slides were washed two times with 400 µl wash buffer. Incubation with monoclonal anti-rat secondary antibody conjugated with FITC and monoclonal anti-mouse secondary antibody conjugated with DyeLight549 was performed at RT for 1 h. Cells were rinsed two times in 400 µl wash buffer. Three hundred microliters of the diluted DAPI solution was added to the cells and the chamber slides were incubated for 5 min at room temperature. Cells were rinsed once with 1X PBS and once with water. The coverslips were removed from the wells and the wells were blotted to remove excess water. One drop of mounting anti-fade medium was dispensed onto the microscope slide per coverslip. Cells were visualized using confocal laser scanning with an Olympus FLUOVIEW FV1200 microscope. Image acquisition was done using the PLAPON60xOSC2 (1.4 NA) objective and the FV10-ASW software. Images were obtained using channel mode (three channels: 405/488/543 nm excitation). Suitable slit width, positions, and other settings were determined for the selected fluorescent dyes by the software.

Dark-field images were captured on an Olympus BX-43 microscope using a CytoViva enhanced dark field condenser and the CytoViva Dual Mode Fluorescence module (CytoViva, Melbourne, USA). All images were taken at 60× or 100× using immersion fluorite objectives and a triple-pass filter configuration.

### Transmission electron microscopy

HCT-116 and RKO (control and treatment) cell lines were centrifuged and the culture medium above the pellets was replaced with 2.7% glutaraldehyde (Electron Microscopy Sciences, Hatfield, USA) in 0.1 M phosphate buffer, pH 7.4. Prefixation was performed at 4 °C for 2 h. The pellets were washed four times with 0.1 M phosphate buffer, and then postfixed for 24 h at 4 °C with 1.5% OsO_4_ (Sigma-Aldrich) in 0.15 M phosphate buffer, pH 7.4. The cells were embedded in EMBed-812 (Electron Microscopy Sciences, Hatfield, USA), after a previous dehydration in an acetone series. Polymerization of the resin was performed at 60 °C for 72 h. Ultrathin sections were cut with a DiATOME diamond knife (DiATOME, USA) on a Bromma 8800 ULTRATOME III ultramicrotome (LKB, Sweden). They were collected on 300 mesh copper grids (Agar Scientific Ltd., Stansted, UK) and double contrasted with saturated alcoholic uranyl acetate (Merck, Darmstadt, Germany) for 12 min, and 2.8% lead citrate (Fluka AG, Buchs, Switzerland). The sections were examined on a JEOL JEM 1010 transmission electron microscope (JEOL Ltd., Japan) at 80 kV, and images were captured using a Mega VIEW III camera (Olympus, Soft Imaging System, Germany).

### Statistical analysis

Evaluation of statistically significant differences was done by using Student’s two-tailed *t*-tests, where a *P* < 0.05 was considered statistically significant. Survival differences within different groups of colon cancer patients were assed using a Kaplan–Meier survival plot and Log-rank (Mantel–Cox) test.

## Electronic supplementary material

Supplementary Figure 1

Supplementary Figure 2

Supplementary Figure 3

Supplementary Table 1

Supplementary figure captions
