# Supplementary figures and images for: The silent healer: miR-205-5p up-regulation inhibits epithelial to mesenchymal transition in colon cancer cells by indirectly up-regulating E-cadherin expression
Source: Cell Death Dis. 2018 Jan 19;9(2):66. doi: 10.1038/s41419-017-0102-8 (PMC5833765; doi:10.1038/s41419-017-0102-8)

## Slide 1
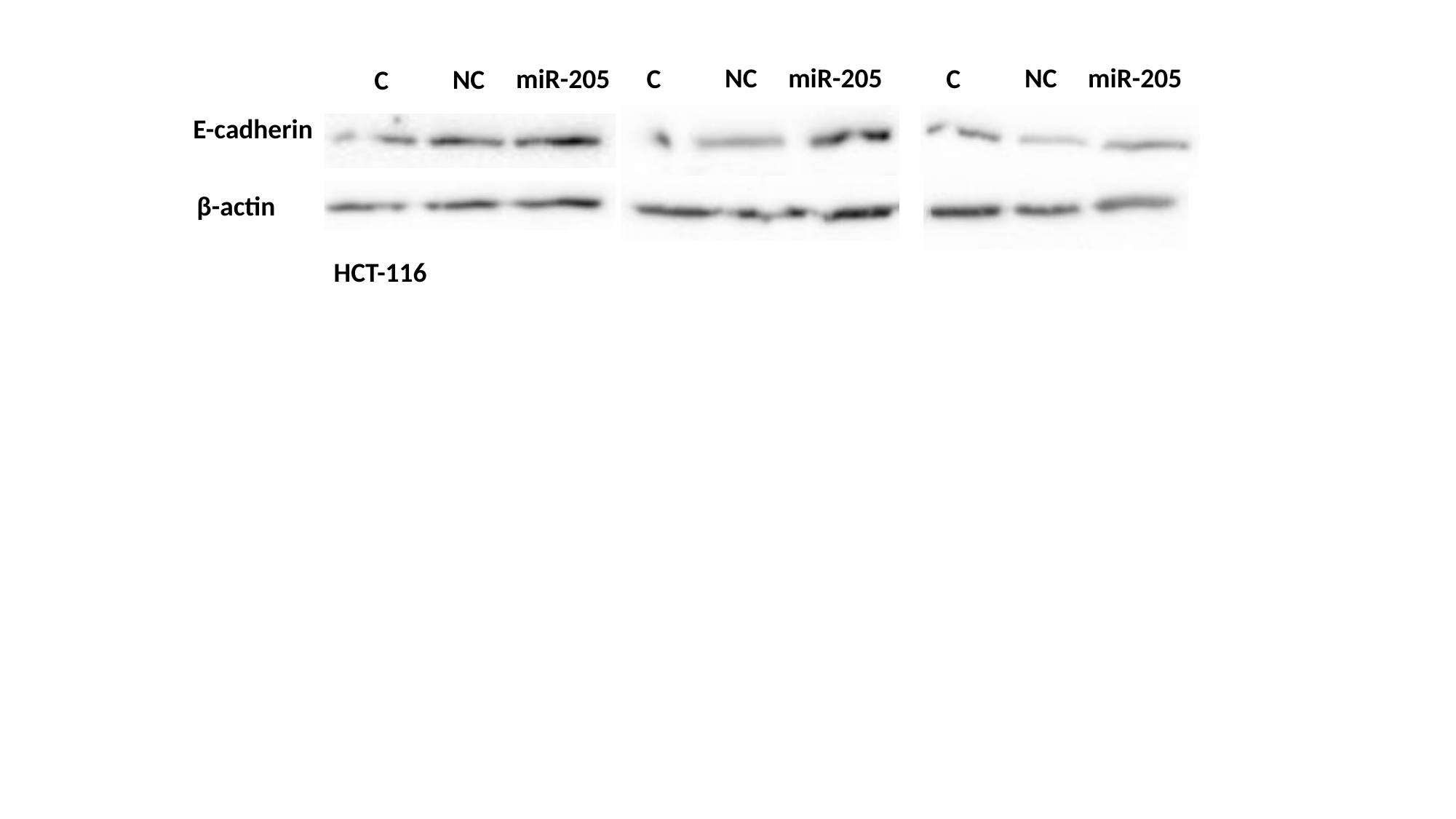

miR-205
miR-205
NC
NC
C
C
miR-205
NC
C
E-cadherin
β-actin
HCT-116

Supplement: Supplementary file 1 — Supplementary Figure 1 [file 41419_2017_102_MOESM1_ESM.pptx]

## Slide 1
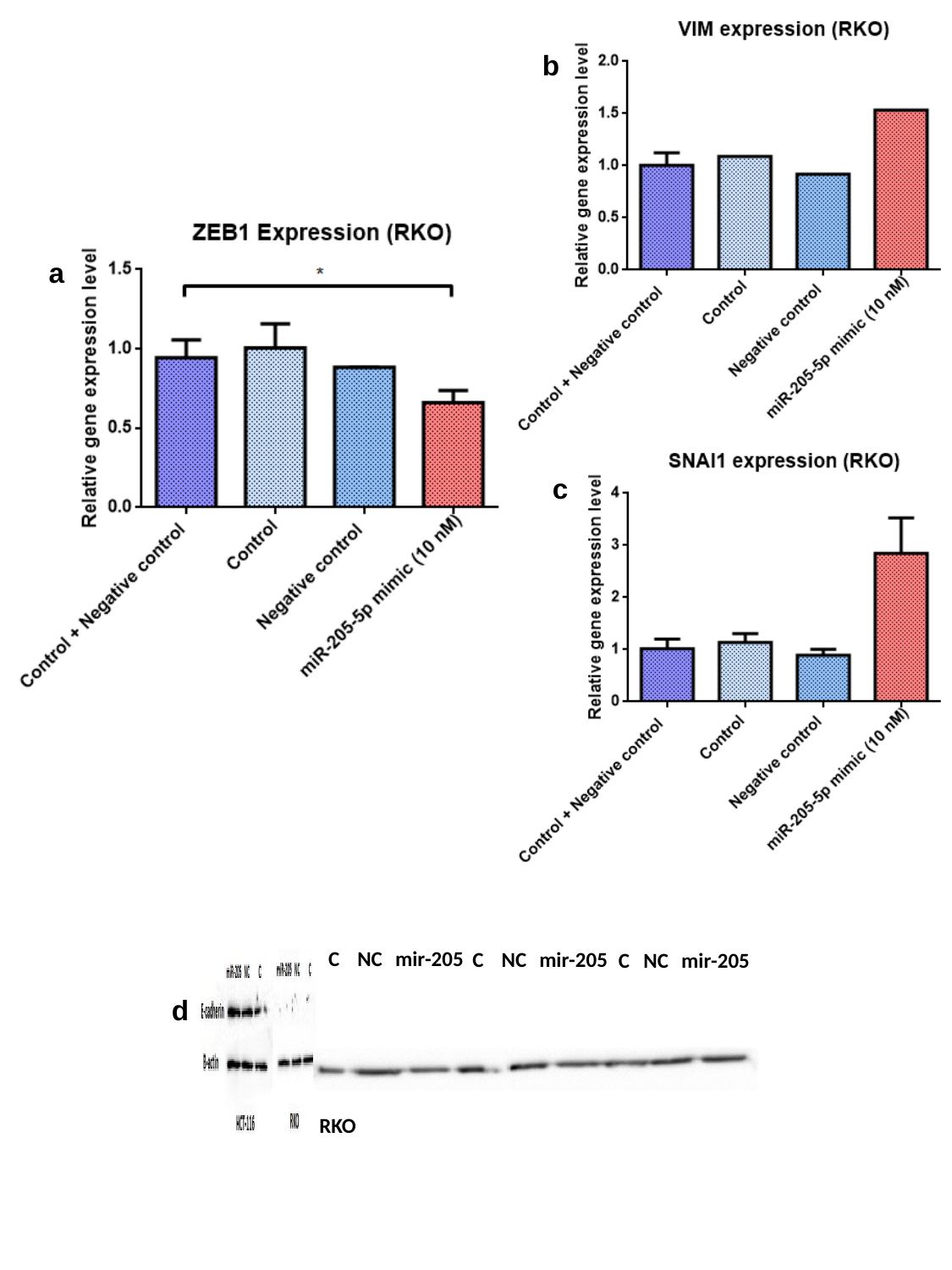

b
a
c
NC
mir-205
C
NC
mir-205
C
NC
mir-205
C
d
RKO

Supplement: Supplementary file 2 — Supplementary Figure 2 [file 41419_2017_102_MOESM2_ESM.pptx]

## Slide 1
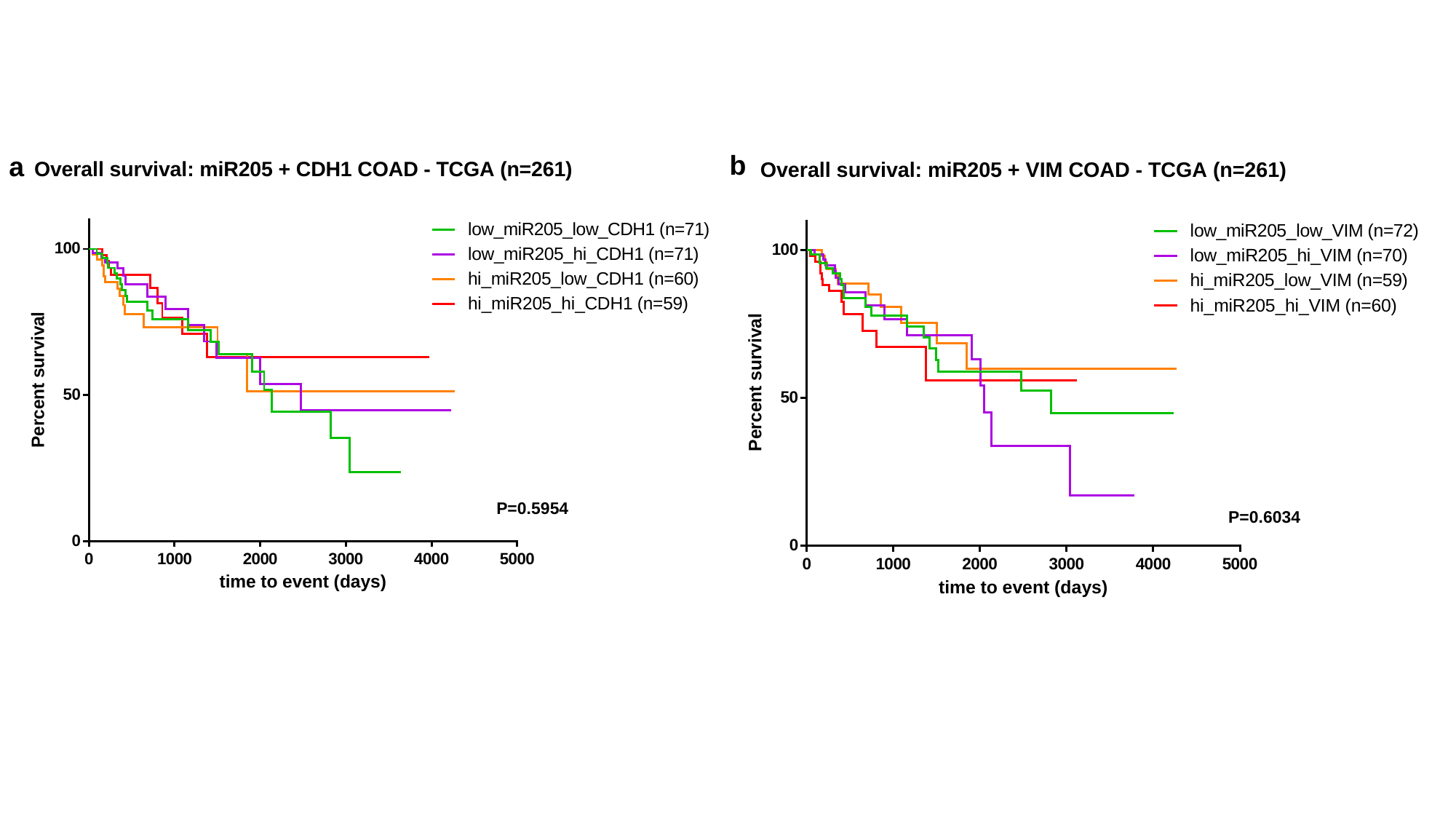

b
a
P=0.5954
P=0.6034

Supplement: Supplementary file 3 — Supplementary Figure 3 [file 41419_2017_102_MOESM3_ESM.pptx]
