# Supplementary material for: The silent healer: miR-205-5p up-regulation inhibits epithelial to mesenchymal transition in colon cancer cells by indirectly up-regulating E-cadherin expression
Source: Cell Death Dis. 2018 Jan 19;9(2):66. doi: 10.1038/s41419-017-0102-8 (PMC5833765; doi:10.1038/s41419-017-0102-8)
Supplement: Supplementary file 5 — Supplementary figure captions [file 41419_2017_102_MOESM5_ESM.docx]

**Supplementary Figure 1**. HCT-116 colon cancer cell line was transfected with either 10 nM human miR-205-5p mimic or negative control (mirVana) using Lipofectamine 2000 and protein levels of E-cadherin (120 kDa) were analyzed 48h post-transfection by WB. All values were normalized to β-actin. Experiments were performed in triplicate

**Supplementary Figure 2.** RKO colon cancer cell line was transfected with either 10 nM human miR-205-5p mimic or negative control (mirVana) using Lipofectamine 2000 and expression values of target and additional genes were analyzed 48h post-transfection by RT-qPCR. All values were normalized to HPRT-1 and RPLP0 housekeeping genes and represented in Grouped Data Graph were on the y axis was plotted the differential expression in fold-change of the analyzed genes compared to control samples. **(a)** RT-qPCR analysis of ZEB1 mRNA expression in HCT-116 cell lines indicated that upregulation of miR-205-5p inhibits the levels of the target gene compared with control, negative control and combination between the two sets of values (data presented as mean ±S.D). Experiments were performed two times in duplicate. RT-qPCR analysis of additional regulatory genes with impact on EMT: **(b)** VIM and **(e)** SNAI1 in RKO cell line transfected with miR-205-5p indicated opposite changes in terms of EMT inhibition, where both mesenchymal markers were found upregulated compared to controls. *CT values for CDH1 and TNF was found as undetermined at a RT-qPCR analysis performed for 40 cycles (identical conditions as for ZEB1, VIM and SNAI1). **(d)** WB for E-cadherin (120 kDa) in RKO colon cancer cell line normalized to β-actin.

**Supplementary Figure 3.** (**a**) Kaplan–Meier analysis of survival between colon cancer patients from TCGA with different combinations of miR-205 and CDH1 expression indicates that association between high levels of miR-205 and CDH1 represents the best molecular pattern in terms of survival. (**b**) Combination of miR-205 and VIM expression is inversely correlated, where high miR-205 and low VIM levels are associated with the best prognosis. Also, elevation of miR-205 levels can partially counteract the negative survival percent associated with increased VIM expression (high miR-205, high VIM vs low miR-205, high VIM)
